# A Multipopulation Dynamic Adaptive Coevolutionary Strategy for Large-Scale Complex Optimization Problems

**DOI:** 10.3390/s22051999

**Published:** 2022-03-04

**Authors:** Yanlei Yin, Lihua Wang, Litong Zhang

**Affiliations:** 1 Faculty of Mechanical and Electrical Engineering, Kunming University of Science and Technology, Kunming 650500, China; yinyanlei1990@163.com; 2 College of Mechanical and Electrical Engineering, Nanjing University of Aeronautics and Astronautics, Nanjing 210016, China; zlt_2015@163.com

**Keywords:** large-scale complex optimization, dynamic adaptive evolutionary network, collaborative topology, search rules

## Abstract

In this paper, a multipopulation dynamic adaptive coevolutionary strategy is proposed for large-scale optimization problems, which can dynamically and adaptively adjust the connection between population particles according to the optimization problem characteristics. Based on analysis of the network evolution characteristics of collaborative search between particles, a dynamic adaptive evolutionary network (DAEN) model with multiple interconnection couplings is established in this algorithm. In the model, the swarm type is divided according to the judgment threshold of particle types, and the dynamic evolution of collaborative topology in the evolutionary process is adaptively completed according to the coupling connection strength between different particle types, which enhances the algorithm’s global and local searching capability and optimization accuracy. Based on that, the evolution rules of the particle swarm dynamic cooperative search network were established, the search algorithm was designed, and the adaptive coevolution between particles in different optimization environments was achieved. Simulation results revealed that the proposed algorithm exhibited a high optimization accuracy and converging rate for high-dimensional and large-scale complex optimization problems.

## 1. Introduction

Many scientific and engineering application problems are complex multi-objective optimization problems involving more decision variables and optimization objectives, such as management and optimal distribution of energy resources [1], the short-term load forecast of power systems [2], the solution time of the joint energy-reserve market clearing problem [3], and wind signal prediction [4], etc. However, in the face of the characteristics of data hybridity in complex problems, it is difficult to use the model-driven method to establish accurate models based on prior knowledge, which has essential limitations. At the same time, the traditional method is difficult to adapt to the uncertainty changes of the search environment and the problem itself in the process of solving complex optimization problems. In particular, with the increase of the dimension of the optimization problem, the search space expands exponentially, and the probability of finding the optimal solution decreases exponentially, which leads to the performance of the algorithm deteriorate sharply. For example, in [5], the trust-tech methods, consensus-based PSO (particle swarm optimization), and local optimization methods that are integrated to compute the small-dimension benchmark optimization problems. It is shown in [6] that the quasi-opposition-based learning (QOBL) and chaotic local search (CLS) strategies with SOS (symbiotic organisms search) are integrated to deal with the global optimization problems with a higher quality solution and faster convergence. The authors of [7] proposed to use the repulsive force rule in mimicry physics to keep the diversity of particles and improve the global search ability of the algorithm. That is, the traditional algorithm mainly enhances the global search ability by improving the diversity of population particles, but it is difficult to solve the high-dimensional complex optimization problems. Consequently, large-scale optimization algorithms have become a research focus in the fields of science and engineering.

In recent years, domestic and foreign scholars have mainly conducted research on two aspects for large-scale optimization algorithms. On the one hand, large-scale complex problems are decomposed into lower-dimensional simple problems in order to get a good solution in a reasonable time. On the other hand, it is a nongrouping strategy, which is mainly solved by utilizing new evolutionary algorithms or adding a local search strategy and tabu search strategy to the original algorithm based on the characteristics of the large-scale and complex problems. Han et al. proposed a dynamic coevolutionary strategy, which integrates the dynamic coevolution mechanism of two probability models and the best individual inheritance strategy into the compact genetic algorithm [8]. For the nongrouping strategy, Aminbakhsh, S. et al. [9] utilized an adaptive differentiation evolution operator in order to solve the local optimization of subproblems, and introduced random search mechanisms based on simulated annealing to improve the global searching capability of the algorithm. Liang, J. [10] reported a random dynamic coevolutionary strategy, which was introduced into the dynamic multi-group PSO algorithm in order to realize the dual grouping of population particles and decision variables. Yao Yucheng et al. [11] made use of the repulsive force rules in pseudo-physics in order to keep the particles diverse and improve the algorithm’s global searching capability. When the population enters the global optimal solution region, the gravitational effect is enhanced and the repulsive effect is reduced. The algorithm’s local searching capability can be improved by using the gravitational effect of particles with better adaptability and global searching capability. Kyle Robert Harrison et al. [12] proposed a parameter-free PSO algorithm based on the prediction model built by machine learning. Moreover, the dynamic grouping strategy and dynamic topology evolution are used to solve large-scale optimization problems. In the latest work [13], a stochastic dynamic coevolution strategy is proposed, which is added to the dynamic multipopulation particle swarm optimization algorithm to realize the double grouping of population particles and decision variables, and thus improves the local search ability and population diversity of the algorithm. The authors of [14] proposed a hybrid topology mixed with fully connected topology and ring topology, where it enables the particles to have stronger exploration ability and fast convergence rate at the same time. However, the above methods rarely aim at high-dimensional complex optimization problems. According to the coupling connection strength between different types of particles, the cooperation relationship and strength between particles are adjusted adaptively in order to improve the algorithm’s adaptability to the complex and variable optimization environment, and thereby overcome the algorithm’s huge space–time cost in solving large-scale complex optimization problems.

Thus, this paper will study the dynamic adaptive coevolution strategy for high-dimensional complex optimization problems, where particles can be divided into model particles, which can guide the whole population to evolve toward the optimal value direction, and ordinary particles, which can guide the population to explore new search directions. According to the cooperation weight between particles and the connecting nodes’ response degree, the two kinds of particles continuously adjust their node connection strength in order to complete the population’s evolution from fully-connected topology at the early stage of evolution to the ring-like topology at the later stage. Based on analysis of the network evolution characteristics of collaborative search between particles, a dynamic adaptive evolutionary network (DAEN) model with multiple interconnections coupling is established in this algorithm. In the model, the swarm type is divided according to the judgment threshold of particle types, and the dynamic evolution of collaborative topology in the evolutionary process is adaptively completed according to the coupling connection strength between different particle types, which enhances the algorithm’s global and local searching capability and optimization accuracy.

The contribution of the paper is the adaptive adjustment of evolutionary topology according to particle node connection strength at different stages of evolution. In particular, the abundant evolution rules are indeed beneficial to solving large-scale complex optimization problems such as sensor network node deployment, acceleration sensor dynamic compensation, sensor optimal configuration, and so on. For example, particle swarm optimization algorithm can be applied to the layout of sensor network nodes, and the global optimization ability of particle swarm optimization algorithm is used to optimize the network coverage. Particle swarm optimization algorithm can also be used for dynamic compensation of an acceleration sensor to expand its frequency range to meet the needs of dynamic measurement. In addition, in order to improve the accuracy of test results in dynamic testing, a particle swarm optimization algorithm can optimize the configuration of sensors, determine the optimal number of sensors, and configure them in the optimal position.

This paper is organized as follows: the problems to be studied are stated in Section 2. The DAEN model and undirected weighted DAEN evolution rules for coevolutionary particle swarm optimization are defined in Section 3. Large-scale complex optimization experiments are given in Section 4 and conclusion are made in Section 5.

## 2. Description of Large-Scale Complex Optimization Problems

Large-scale complex optimization problems are often nondifferentiable and nonlinear. When solving large-scale complex optimization problems with continuous iterations, dimension disaster is likely to be encountered [15]. In order to overcome the algorithm’s huge computational time and space cost in solving the large-scale complex optimization problems, the algorithm’s optimization accuracy, convergence speed, and solution success rate in large-scale complex optimization problems have been improved. The large-scale complex optimization problem is expressed by the following formula:

(1) $$\{\;\;\begin{array}{c} \min\;f\left(x\right)\;/\;\max\;f\left(x\right)\\ X_{i}=\left(x_{i1},x_{i2},\cdots ,x_{iD}\right)\\ s.t.\in \Omega \end{array}.\;$$

where min f(x)/max f(x) refers to the objective function of the optimization problem. In the single objective optimization problem, it can be understood as a real valued continuous nonlinear objective function mapping from d-dimensional space to one-dimensional fitness value. $X_{i}=\left(x_{i1},x_{i2},\cdots ,x_{iD}\right)$ is the boundary constraint. *D* is the number of decision variables, that is, the dimensions of the optimization problem. In large-scale setting, the number of decision variables *D* is generally greater than 100, usually reaching more than 1000 dimensions, and $x_{i1}$ is the decision variable.

## 3. Coevolutionary Particle Swarm Optimization Algorithm Based on DAEN

With the increase of the dimensions of large-scale optimization problems, the time-varying law presents multiscale characteristics. If the full connection method is adopted, it is easier to fall into local optimization, and the performance of particle swarm optimization algorithm will degrade rapidly, so it is difficult to directly apply this method to large-scale complex optimization problems. To solve this problem, we need to improve and expand the population optimization model, and establish information interaction and association rules between different search tasks and cooperative populations.

**Figure 1:**
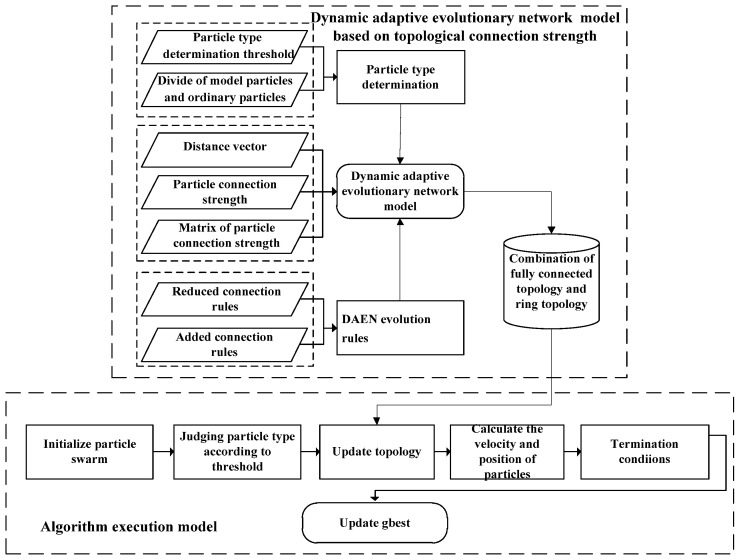
Flowchart of the algorithm.

Specifically, the coupling degree of evolution within and between communities can be reduced through the mechanism of coevolution, and the node strength is used to represent the cooperation strength among communities. In particularly, the collaborative rules are used to trigger the multicommunity collaborative search process. And thus, the scalability and adaptability of the algorithm are improved through the dynamic reorganization of the cooperation relationship. On the other hand, the parallel implementation mechanism is adopted to set up the global optimal location storage area of community members, which could complete the asynchronous iteration of each search process. Moreover, the iterative results of each step are sent to other processes in the form of broadcast to reduce process communication and improve the optimization efficiency of the algorithm effectively. Consequently, the DAEN model and undirected weighted DAEN evolution rules for coevolutionary particle swarm optimization are presented in this section. The coevolutionary flowchart is shown in Figure 1.

1. Dynamic adaptive evolutionary network model based on topological connection strength. It is well-known that a network can be regarded as the combination of vertex set and edge set. Thus, we have used the edge to represent the connection between particles, which can describe the cooperative search relationship between particles, and analyze its adaptive cooperative evolution law. Following this idea, the particles were divided into model particles and ordinary particles according to the threshold value of particle type, where the model particles have strong local optimization ability, and ordinary particles have strong global exploration ability. On this basis, the topological connection relationship between different particles was established, and the cooperation and optimization ability of particles were comprehensively evaluated by the distance vector and connection strength between particles, where the evolution rules of topological connection among particles were formulated to form a self-adaptive evolutionary network model that adapts to the environmental changes of large-scale complex optimization problems.
2. Algorithm execution model. During the algorithm execution, the topology connection relationship among particles was adaptively adjusted according to the complex search environment, and the current optimal location and global optimal location storage area was set. Thus, the new global optimal position obtained was sent to other processes in the form of broadcast of the asynchronous iteration process, which was calculated as the current generation global optimal value. Consequently, the process communication could be reduced, and the optimization efficiency of the algorithm was improved while conforming to the biological mechanism of particle swarm optimization.

### 3.1. Standard Particle Swarm Algorithm

The PSO algorithm was inspired by social animals, such as flocks of birds and fish. PSO is initialized by a set of random solutions, which searches for the optimal solution through generation updates [16].

There are m particles in D dimension search space. The particle i, the position of i=1, 2, ⋯, m being $X_{i}=\left(x_{i1},{\;x}_{i2},\;\cdots ,{\;x}_{iD}\right)$, experiences the optimal position, which is recorded as $P_{i}=\left(p_{i1},\;p_{i2},\;\cdots ,\;p_{iD}\right)$, also known as particle extremum (pbest). The best position that all particles in the population have experienced is $P_{g}=\left(p_{g1},\;p_{g2},\;\cdots ,\;p_{gD}\right)$, also known as global extreme (gbest). Particle velocity is expressed in terms of $V_{i}=\left(v_{i1},\;v_{i2},\;\cdots ,\;v_{iD}\right)$. For each generation, the particles update themselves by tracking two extremes, that is, the particles evolve according to the following formula.

(2) $$v_{id}^{t+1}=\omega \cdot v_{id}^{t}+c_{1}\cdot rand_{1}\left(\;\right)\cdot \left(P_{id}^{t}-x_{id}^{t}\right)+c_{2}\cdot rand_{2}\left(\;\right)\cdot \left(P_{gd}^{t}-x_{id}^{t}\right)$$

(3) $$x_{id}^{t+1}=x_{id}^{t}+v_{id}^{t+1}$$

The above formula describes that in each iteration process, each member particle changes its own state according to the position and speed update rules, and continuously improves itself by tracking the historical optimal value of the particle member and the global optimal value of the community. Where *t* or *t* + 1 is the number of iterations, *ω* is the inertia weight, $c_{1}$ and $c_{2}$ are acceleration constants, and $rand_{1}\left(\;\right)$ and $rand_{2}\left(\;\right)$ are random functions that vary in the range of [0, 1]. The first part is the particles’ searching speed, which reflects the particles’ memory. The second part is the “cognition”, which reflects the particles’ thinking and affirmation. The third part is the “society”, reflecting the information sharing and cooperation among particles. Significantly, each search agent is checked for out-of-search space and amended. If X^*t*+1^ is beyond the upper boundaries of the search space, X^*t*+1^ is the value of the upper boundary. If X^*t*+1^ is beyond the lower boundaries of the search space, X^*t*+1^ is the value of the lower boundary.

### 3.2. DAEN Model

**Figure 2:**
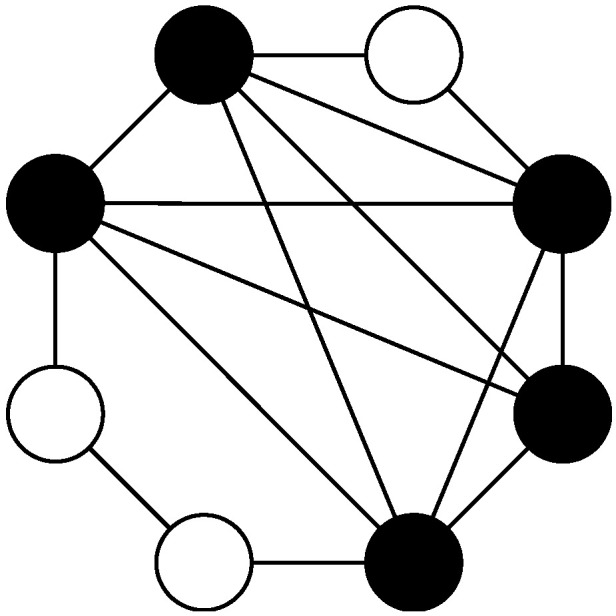
DAEN model.

The standard particle swarm algorithm is a global optimization model based on the optimal particles, whose neighborhood structure is equivalent to a fully-connected network, which can converge to the optimal value more quickly. However, it is inconvenient to use a fully-connected network to process high-dimensional data. For more complex high-dimensional data, the fully-connected method falls more easily into the local optimum. Based on the six-degree separation theory [17] and small-world network [18], the DAEN, with a fast convergence speed and strong global searching capability, is formed by combining the fully-connected topology with the ring topology, as shown in Figure 2. In this topology, there are cooperative relations due to different types of particles, including the cooperative relations between model particles and other model particles, model particles and ordinary particles, and ordinary particles and other ordinary particles.

From a mathematical perspective, the network can be regarded as a combination of vertex sets and edge sets. In order to better describe DAEN and establish its evolution model, the following definitions are given.

**Figure 3:**
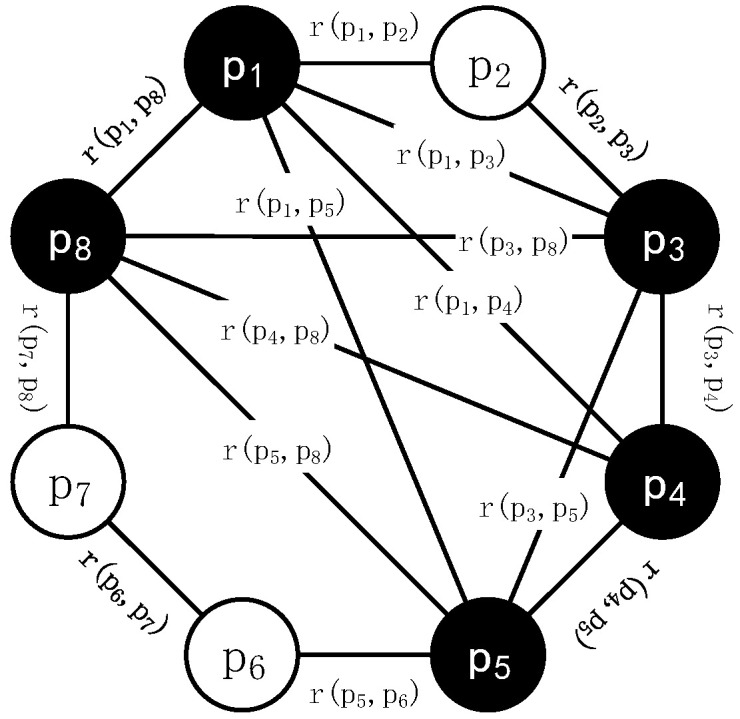
Undirected weighted DAEN model.

**Definition** **1.** *In the DAEN structure, edges between the nodes are undirected and have connection strength. Connections between particles can be represented by the undirected weighted graph* G(P,R)*, as shown in Figure 3.Where* $P=\left(p_{1},p_{1},\ldots ,p_{1},\ldots ,p_{n}\right)$*represents the set of all particles in the population.* $R=\left(r\left(p_{1},p_{2}\right),r\left(p_{1},p_{3}\right),\ldots ,r\left(p_{i},p_{j}\right),\ldots ,r\left(p_{n},p_{n}\right),\right)$*represents the set of connection relations among particles.* $\forall r_{s}\left(p_{i},\;p_{j}\right)\in R,\;s=1,\;2$*, where r_*1*_ represents the connection relationship of the ring topology in the first step of initializing the topology. r_*2*_ represents the connection between the model particles in the second step of initializing topology. |R_i_| denotes the module with connection relation set R with particle p_i_, and indicates that there are |R_i_| edges directly connected with p_i_.*

**Definition** **2.** *Particle type determination threshold *F*.*

(4) $$F=\frac{\sum_{i=1}^{n}f_{i}}{n}$$

*where f_i_ is the fitness value of particle p_i_ and n is the total number of particles in the population.*

According to particle type determination threshold *F*, the particles in the population can be divided into model particles *p_m_* and ordinary particles *p_o_*. If the fitness value, *Fi*, of the particle satisfies *F_i_* < *F*, the particle has a better fitness value, which is divided into model particles in order to guide the whole population to evolve toward the optimal value direction. On the contrary, if *F_i_* ≥ *F*, the particle fitness is poor, and it is divided into ordinary particles in order to guide the population to explore a new direction.

In order to determine whether DAEN needs to add connections or to continue to reduce them, the evaluation index of the optimal value for population nodes $gbest_{i}$ is introduced: distance vector *H*.

**Definition** **3.** *Distance vector. The difference between the global optimal value* $gbest_{i}$*of the population and the individual optimal position* $pbest_{i}$*of m particles with t = n iterations in the population is calculated and absolute values are taken, then the population’s distance vector H under the current iteration times is obtained.*

(5) $$H=\left(h_{1},h_{2},\cdot \cdot \cdot ,h_{m}\right)$$

**Definition** **4.** *Particle connection strength. In DAEN, the two particles’ connection strength is defined as the undirected weighted graph’s weight* $v_{ij}$*. The undirected weighted graph’s connection strength is calculated from the two currently connected particles’ fitness values:*

(6) $$v_{ij}=\{\begin{array}{c} 1-\frac{\left|f_{i}-f_{j}\right|}{f_{best}},\;\forall F\left(r\in r\left(p_{i},p_{j}\right)\right)<F\\ 0,\;\forall F\left(r\in r\left(p_{i},p_{j}\right)\right)\geq F \end{array}$$

Suppose that there are *n* particles in the undirected weighted DAEN, and there are *n* particles with connection relationship *r* with particle *p_i_*_,_ then the local aggregation coefficient of particle *p_i_* is:

(7) $$\mu_{i}=\frac{\sum_{j,k}^{n}r_{jk}}{n\left(n-1\right)}$$

On the basis of Equation (2), the particle connection strength matrix can be expressed as *C*. *E* is the nth order unit matrix, and matrix *C* can be expressed as:

(8) C=v(r(pi,pj))×E

According to Equations (2) and (3), the particle undirected weighted DAEN model can be expressed as follows:

(9) $$M={\left(B,C\right)}_{nm},m=n+1$$

### 3.3. Undirected Weighted DAEN Evolution Rules

**Figure 4:**
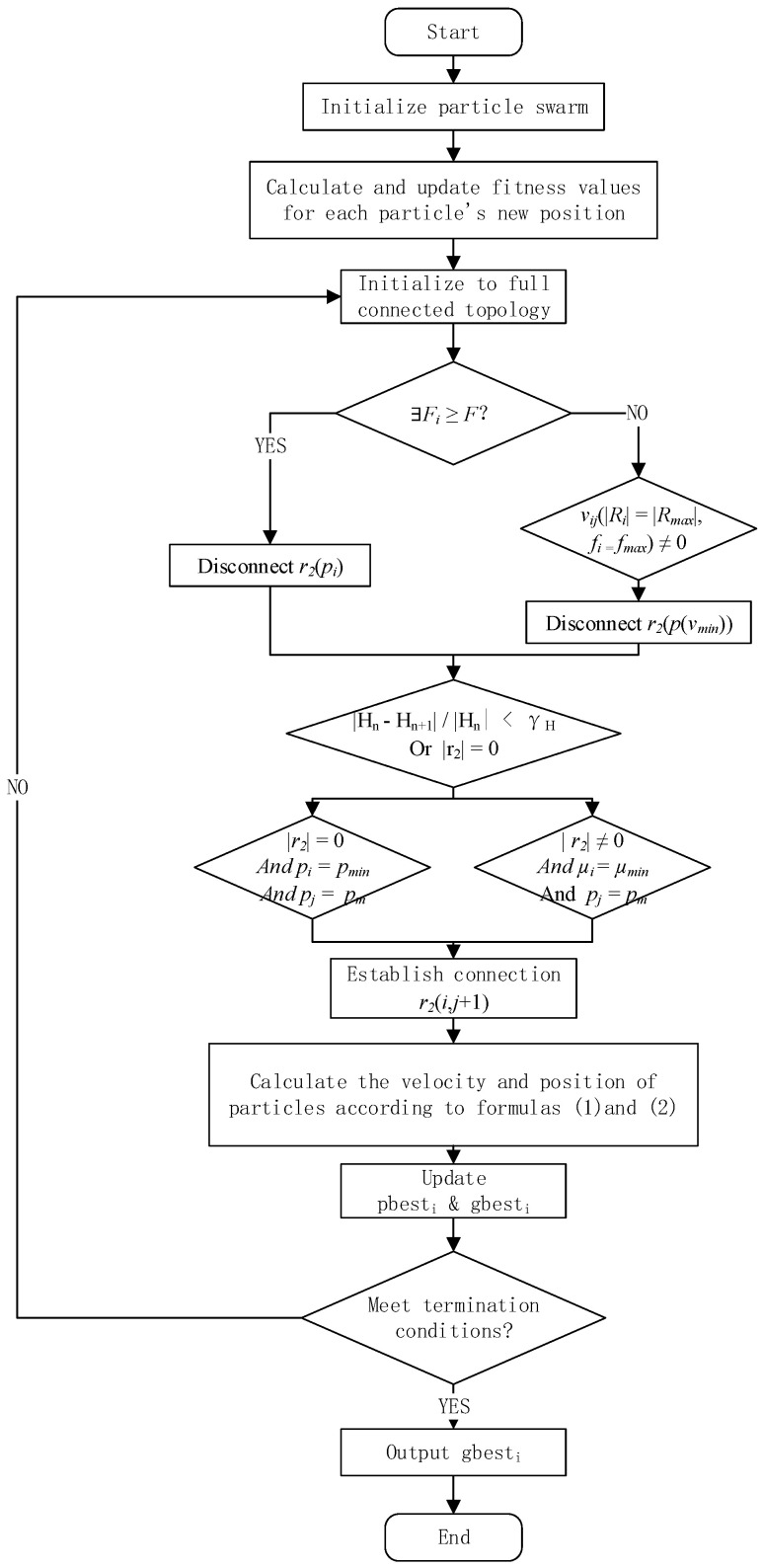
Topological evolution steps.

The calculation of the reduced connection rule and the added connection rule for the undirected edge is based on particle connection strength and particle fitness value. Hopefully, two high-connection strength particles can get more reliable connections, and particles with good fitness values can get more connections. After each iteration, the particles’ fitness value is recalculated and the particle type is judged. Meanwhile, the reduced-connection or added-connection operations are carried out. Figure 4 shows the evolution process.

1. Initialize the topology: initialize particle swarm, set the fitness value threshold, calculate each particle’s fitness value, judge whether the particles’ fitness value reaches the threshold value, and define the particles that reach the threshold value as model particles and those that do not as ordinary particles. That is, the topology is initialized as a ring topology, and the connections between the model particles are fully connected in order to build an initial fully-connected topology.
2. Reduced-connection rule: in order to make the algorithm jump out of local optimization and seek global optimal solution, the reduced connection operation is performed according to the edge’s reduced connection rule every time the algorithm evolves. The fully connected topology’s initial search speed is faster, but it is easy to fall into local optimization. In this paper, two kinds of reduced-connection rules are designed.
  Rule 1: If $\exists F_{i}\geq F$, Then $\forall r_{2}\left(p_{i}\right)=0$;
  Rule 2: If $\forall F_{i}<F,\;v_{ij}\left(\left|R_{i}\left|=\right|R_{max}\right|,\;f_{i}=f_{max}\right)\;\neq 0$, Then $r_{2}\left(p\left(v_{min}\right)\right)=0$;
3. Reduced-connection termination rule: according to the connection relationship *r* between particles and the distance vector, two kinds of reduced-connection termination rules are designed:
  Rule 3: If $\left|\;r_{2}\right|=0$, End;
  When the change of the distance vector’s module for the particle is less than the designed threshold value, the reduced connection is stopped:
  Rule 4: If $\left|H_{n}-H_{n}+1\;\left|\;/\right|H_{n}\;\right|<\gamma H$, End;
4. Added-connection rule: according to the number of $r_{2}$ edges of the model particle *p_i_*, i.e., the size of $\left|\;r_{2}\right|$ and the local aggregation coefficient, the added-connection rules are designed to improve different particles’ adaptability and balance the particles’ global and local searching capability. Two kinds of added connection rules are designed.
  When DAEN is a ring topology, and when $\left|\;r_{2}\right|=0$, the model particle $p_{j}$ with the farthest distance from $p_{i}$ is selected in order to establish the connection:
  Rule 5: If $\left|\;r_{2}\right|=0,\;p_{i}=p_{min}$ and $p_{j}=p_{m}$, Then $r_{2}\left(i,j+1\right)=1,\;j=i+N/2+n\left(n=0,1,2,3\right)$
  When $\left|r_{2}\right|\neq 0$, the local aggregation coefficient μ of all model particles is calculated, and the model particle with the smallest μ is selected in order to establish a connection with the model particles farthest away from the population:
  Rule 6: If $\left|r_{2}\right|\neq 0,\mu_{i}=\mu_{min}$ and $p_{j}=p_{m}$, Then $r_{2}\left(i,j+1\right)=1,\;j=i+N/2+n\left(n=0,1,2,3\right)$.

### 3.4. Algorithm Execution Steps

**Figure 5:**
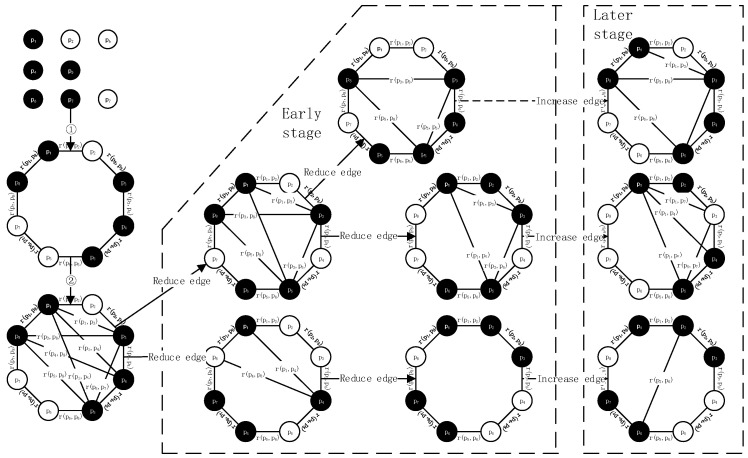
DAEN evolution.

When improving search speed and searching capability, each particle is given subjective initiative, considering the evolutionary method diversity presented by particles with different individual attributes, and resource sharing among members in the community and information interaction between the communities are fully utilized. Based on the particles’ fitness values and connection strength, the added-connection and reduced-connection rules for edges are designed, and the added-connection and reduced-connection operations are performed, As shown in Figure 5. Then, in order to improve search efficiency in the algorithm’s early stage and enhance the local searching capability in the later stage, the DAEMPSO algorithm is proposed by using the DAEN model evolution in order to combine the fully-connected topology with the ring topology.

Based on this parallel idea, the specific pseudo-code for the DAEMPSO algorithm (Algorithm 1) is:
**Algorithm 1**: DAEMPSO.**procedure** DAEMPSO**for** each particle *i*:Initialize velocity *V_i_* and position *X_i_* for particle *i*. Evaluate particle *i* and set *pBest_i_* = *X_i_***end for***gBest* = *min{pBest_i_}***for** *i* = l to neighborhood*F = $\frac{\sum_{i=1}^{n}fitness_{i}}{n}$***if** |*r_*2*_*| ≠ 0, & |*H_n_* − *H_n+*1*_*|/|*H_n_*| > *γ_H_***if** ∃*F_i_* > *F* // Model particleUpdate neighborhood // Reduce edgeUpdate the velocity and position of particle *i*. Evaluate particle *i***if** fitness(*X_i_*) < fitness(*pBest_i_*), *pBesti* = *X_i_* // Update individual optimal value**if** ∀*F_i_* < *F*  //Ordinary particle**if** *v_ij_*(|*R_i_*| = |*R_max_*|, *f_i_* = *f_max_*) ≠0repeat steps 10–12 // Reduce edge**if** |*H_n_* − *H_n+*1*_*|/|*H_n_*| < *γ_H_***if** μ*_i_* = μ*_min_* and *p_j_* = *p_m_*repeat steps 10–12  // Increase edge**if** |*r_*2*_*| = 0**if** *i* = *i_min_* and *j* = *j_m_*repeat steps 10–12  // Increase edge**if** fitness(*pBest_i_*) < fitness(*gBest*), *gBest* = *pBest_i_*;  // Update neighborhood optimum**end for**print *gBest***end producer**

## 4. Analysis of Simulation Results

### 4.1. Test Function and Experimental Environment

In order to analyze the DAEMPSO algorithm’s adaptability, execution efficiency, and calculation accuracy in solving high-dimensional complex problems, 13 high-dimensional complex multimode functions of the virtual simulation library are used for simulation analysis. These functions include unimodal and multimodal functions, and the variable dimensions can be set. The thirteen test functions’ main characteristics are shown in Table 1 and Table 2. The first 13 problems are classical benchmark functions utilized in the optimization literature [19,20,21,22].

### 4.2. Simulations

In the experiment, GWO [23], BOA [24], MPA [25], and COOT [26] were selected to compare with DAEMPSO in order to verify the effectiveness of the new strategy. GWO has achieved good results in large-scale global optimization algorithm, and BOA, MPA, and COOT are three recently proposed large-scale optimization algorithms. Compared with these algorithms, the effectiveness of the DAEMPSO based on coevolution strategy can be verified. Specifically, the GWO algorithm mimics the leadership hierarchy and hunting mechanism of grey wolves in nature. Four types of grey wolves, such as alpha, beta, delta, and omega are employed for simulating the leadership hierarchy. BOA is mainly based on the foraging strategy of butterflies, which imitates their sense of smell to determine the optimal value of the function. According to the motion type and velocity of the predator, MPA has an optimal motion strategy for the predator to maximize the encounter rate with the prey. The Coot algorithm imitates the movement patterns of two different birds on the water surface: in the first stage, the movement of birds is irregular, and in the second phase the movements are regular. At the same time, the colony moves to a group of leaders to obtain food supply, and the movement of the end of the colony is in the form of a chain of coots, each coot moving behind the coots in front of it.

The parameters of the five algorithms are set as follows. That is, the dimensions are 500, 800, and 1000 and the maximum number of iterations is 500. The above algorithms are run independently 25 times, and the optimal value, average optimal value, and success rate are recorded. Table 3, Table 4, Table 5, Table 6, Table 7 and Table 8 shows the test results.

As compared with Table 3, Table 4, Table 5, Table 6, Table 7 and Table 8, when the dimension is set to 500, 800, and 1000 for high-dimensional complex optimization functions, each optimization algorithm can better adapt to the peak shape changes of F1 and F6 with the increase of the search area, but it has poor adaptability for multimodal functions like F7, F12, and F13. That is, the number of peaks of the function has a great impact on the algorithm convergence. It is shown that GWO has a local convergence for high-dimensional functions. In particular, BOA has worse local convergence for F4, F5, F7, F12, and F13. Analysis of the reasons posits that BOA did not consider the typical characteristics of large-scale optimization problems; although BOA can divergent the search path in the search process, it is difficult to jump out of multiple local optimal points of the high-dimensional multimodal functions or high-dimensional unimodal functions, which leads to the poor performance of the algorithm in solving large-scale optimization problems. Because the search process in MPA uses a phased strategy, the search stages cannot be dynamically divided, which leads to poor performance in the testing process of high-dimensional multimodal functions. COOT does not have the previous speed parameter in the proposed algorithm, and the location of each search agent is updated according to the location of the current search agent and the location of multiple search agents. On the other hand, the proposed algorithm updates a new position based on topological link motion and random motion in different directions, and it can converge to the optimal value in most cases. It is noted that DAEMPSO can adaptively adjust the evolutionary topology according to particle node connection strength at different stage of evolution, which evolves between the fully-connected topology and the ring topology by evolutionary rules for different optimization environments. Consequently, the optimization accuracy of DAEMPSO is significantly higher than that of the above four optimization algorithms.

**Figure 6:**
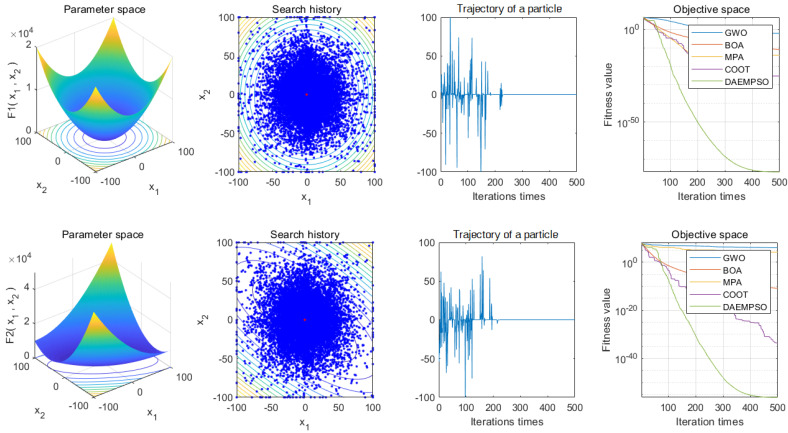

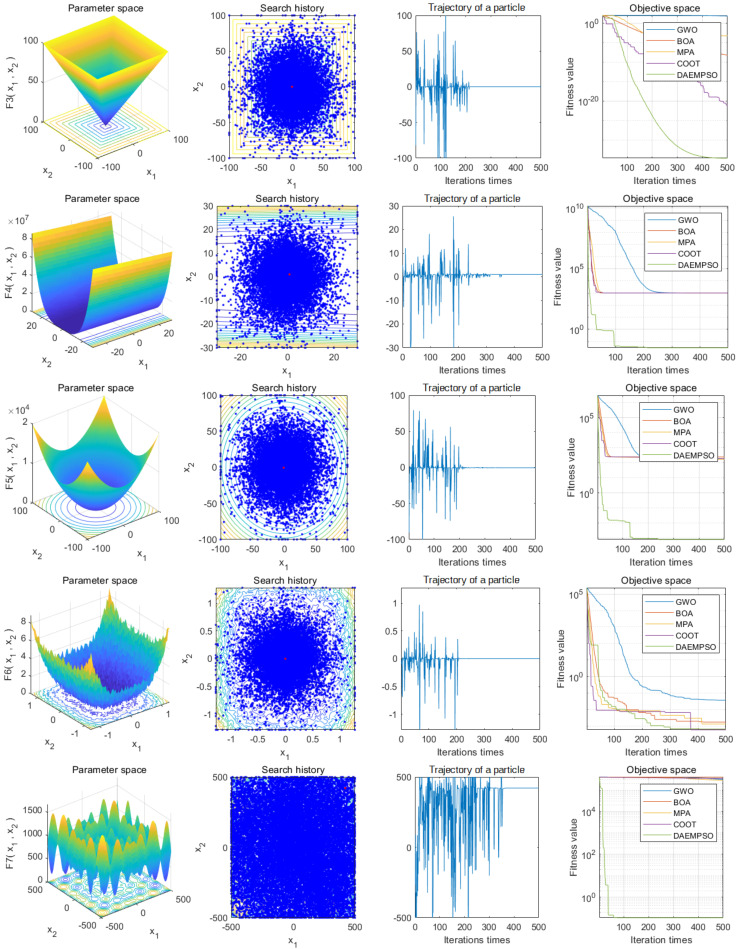

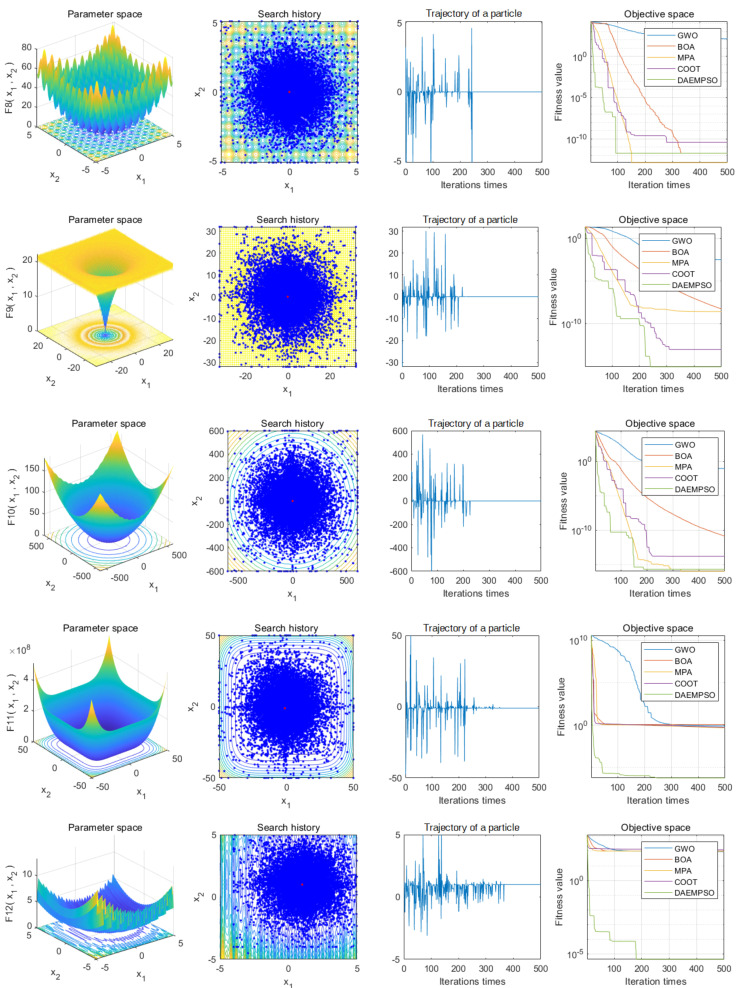

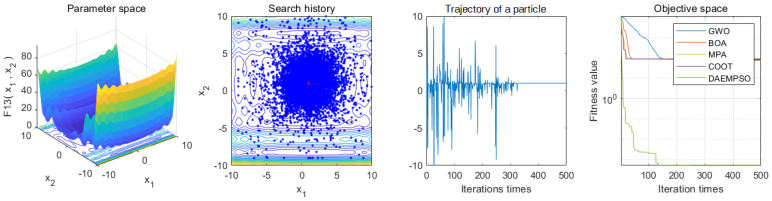
Convergence curves of five optimization algorithms for function (1000-dimension) F1–F13.

**Figure 7:**
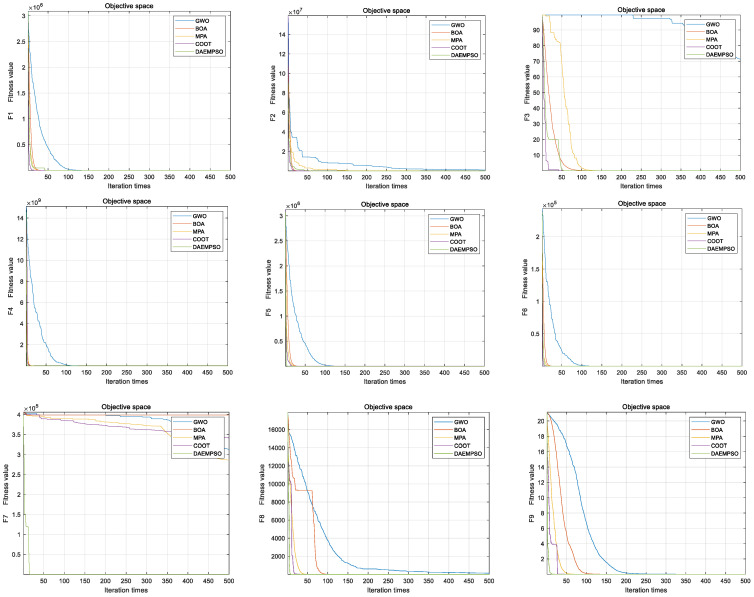

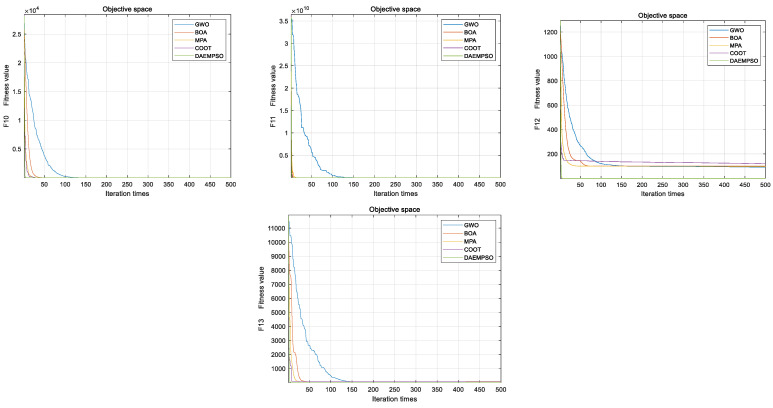
Convergence curves of five optimization algorithms for function (1000-dimension) F1–F13.

The test function is a large-scale global optimization algorithm test function set, which contains single-mode and multi-mode characteristics. From the results shown as Figure 6 and Figure 7, we can see the effectiveness of the DAEMPSO algorithm in solving large-scale optimization problems, which are determined by the characteristics of dynamic topology connection based on performance evaluation of particle collaboration. It divides the population into model and ordinary particles, and the two kinds of particles continuously adjust their node connection strength in order to complete the population’s evolution from fully-connected topology at the early stage of evolution to the ring-like topology at the later stage. Noticeably, in test functions F4, F12, and F13, both BOA and MPA algorithms use the coevolutionary strategy of population grouping, where due to the strong local search ability of dynamic multigroup strategy and the sacrifice of global search ability, the convergence ability of the algorithm is not strong, so the test results of BOA and MPA are not good. Moreover, COOT converges in multiple test functions, but fails to converge at F7, F12, and F13. Generally, when the dimension is 1000, the above algorithm’s convergence performance is similar for F1, F6, F9, and F10, the convergence speed is faster for F7, F9, and F10. But for F5, F7, F12, and F13, the performance of DAEMPSO is obviously better than other algorithms, in which the evolutionary topology can be adjusted adaptively according to the connection strength of particle nodes, and rich evolution rules are formulated considering the characteristics of large-scale complex optimization problems during the implementation of the algorithm.

### 4.3. Statistical Analysis of DAEMPSO

**Figure 8:**
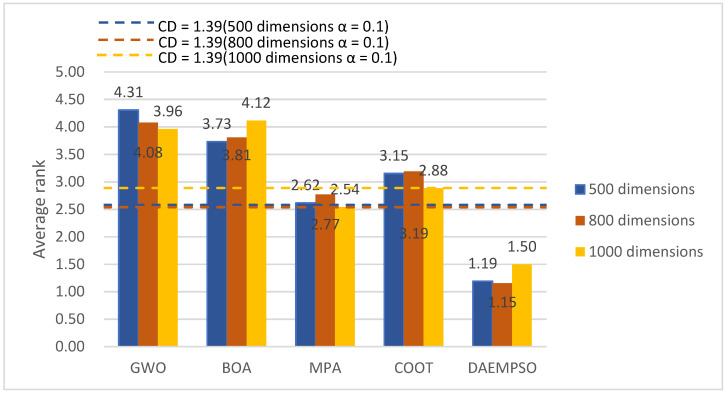
Bonferroni–Dunn’s test for different methods and groups with α = 0.1.

This section uses the Bonferroni–Dunn test to analyze the competitiveness of DAEMPSO with respect to its other competitors. In order to have a reliable test, this study categorized the inspection data into three groups. The three groups of data are the basic test functions of different algorithms in 500, 800, and 1000 dimensions, which are ranked according to the running results of the convergent average. This test demonstrates that there is a significant difference in performance between two algorithms if the difference in average ranking of methods is greater than the critical difference (CD). Figure 8 shows the average ranking of methods in different dimensions with a significance level of 0.1. DAEMPSO can significantly outperform those algorithms, whose average ranking is above the threshold line shown in the figure in 500 and 800 dimensions. The threshold line of each group is identified by its color. As is observable from the figure, DAEMPSO is ranked first and has significant advantages over other algorithms.

### 4.4. Result Analysis

As the aforementioned simulation results, one can observe that the DAEMPSO algorithm shows significantly superior convergence performance for multidimensional F1–F6 and F7–F13 in comparison to other improved methods such as GWO, BOA, MPA, and COOT. On the other hand, it is noted that the other aforementioned methods may fail to retain their convergence speed with the increasing dimensions. In particular, as shown in Figure 6 and Figure 7, the presented method can guarantee a well-balanced performance for the exploratory and exploitative propensities on problem’s topographies with high dimensions. Moreover, these comparative results show even worse ability between several methods such as the GWO, BOA, MPA, and COOT, with high-quality solutions found by DAEMPSO. Consequently, the dynamic coevolution behaviors are of great importance for the high-dimension problems. To address this issue, some efforts have been advanced to exploit the ability of adaptive dynamic topology evolution in different evolution stages in Table 3, Table 4, Table 5, Table 6, Table 7 and Table 8. It is shown that DAEMPSO is validated to adjust node connection strength to guarantee the evolutionary topology adaptively in different dimensions. The results also support the superior exploratory strengths of DAEMPSO for multimodal and hybrid composition landscapes. Moreover, the results for 1000 dimensions functions in Table 3, Table 4, Table 5, Table 6, Table 7 and Table 8 also disclose that the improved convergence performance can be achieved for the proposed algorithm in comparison to other conventional methods.

The following features are provided to demonstrate the efficacy of the proposed methods:

1. Division of superior and inferior populations with regard to the average location of particles can encourage the exploratory behavior of DAEMPSO in the initial iterations.
2. Node connection strength has a dynamic randomized time-varying nature to guarantee the adaptive adjustment of DAEMPSO exploration and exploitation patterns.
3. Different topological evolution patterns according to the connection strength of particle nodes enhance the exploitative behaviors of DAEMPSO when performing a local search.
4. The progressive topological coevolution scheme can be used to drive the model particles to find the optimal position step by step, so as to improve the quality of the solution and enhance the iterative ability of the algorithm.
5. A series of adaptive adjustment strategies, based on H and C for the DAEN model can inspire particles to select the best topological link relationship. Such ability also has a constructive impact on the exploitation potential of the algorithm.

## 5. Conclusions

In this paper, a dynamic adaptive coevolutionary strategy is proposed for large-scale complex optimization problems, where particles can be divided into model particles and ordinary particles. Thus, the model particles can guide the whole population to evolve toward the optimal value direction, the ordinary particles can guide the population to explore new search directions. According to the cooperation ability between particles, the two kinds of particles continuously adjust their node connection strength in order to complete the population’s evolution from fully-connected topology at the early stage of evolution to the ring-like topology at the later stage. The contribution of this paper is to adjust the evolutionary topology adaptively in different evolution stages according to the connection strength of particle nodes. The dynamic evolution of connection topology can solve the problem of multiple decision variables and correlation among variables, while population dynamic grouping can solve the problem of multimodality and algorithm convergence too fast and fall into a local optimum. Finally, the proposed algorithm is compared with other algorithms in benchmark function set testing to verify the effectiveness of the results. However, there are still some problems to be solved in future work.

1. Parameter adjustment: the new algorithm does not discuss the parameter adjustment to increase the adaptive mechanism of parameters and reduce the complexity of the algorithm.
2. Practical application: the algorithm proposed in this paper has good results on the test platform, but the results in practical application have not been verified, so the effectiveness of the algorithm in practical optimization problems such as large-scale production line collaborative operation needs to be verified.

## Figures and Tables

**Table 1:** High-dimensional unimodal benchmark test functions.

	Function Name	Function	Dimensions	Search Space	Theory Optimum
F1	SPHERE FUNCTION	$f\left(x\right)=\overset{n}{\underset{i=1}{\sum }}x_{i}^{2}$	1000	[−100, 100]	0
F2	ROTATED HYPER- ELLIPSOID FUNCTION	$f\left(x\right)=\overset{n}{\underset{i=1}{\sum }}\left(\overset{i}{\underset{j-1}{\sum }}x_{j}^{2}\right)$	1000	[−100, 100]	0
F3	SCHWEFEL’ S PROBLEM	$f\left(x\right)=max_{i}\left\{\left|x_{i}^{2}\right|,1\leq i\leq n\right\}$	1000	[−100, 100]	0
F4	ROSENBROCK FUNCTION	$f\left(x\right)=\overset{n-1}{\underset{i=1}{\sum }}\left[100{\left(x_{i+1}-x_{i}^{2}\right)}^{2}+{\left(x_{i}-1\right)}^{2}\right]$	1000	[−30, 30]	0
F5	STEP FUNCTION	$f\left(x\right)=\overset{n}{\underset{i=1}{\sum }}{\left(\left[x_{i}+0.5\right]\right)}^{2}$	1000	[−100, 100]	0
F6	QUARTIC FUNCTION	$f\left(x\right)=\overset{n}{\underset{i=1}{\sum }}ix_{i}^{4}+random\left[0,1\right]$	1000	[−1.28, 1.28]	0

**Table 2:** High-dimensional unimodal benchmark test functions.

	Function Name	Function	Dimensions	Search Space	Theory Optimum
F7	SCHWEFEL FUNCTION	$f\left(x\right)=418.9829d-\overset{d}{\underset{i=1}{\sum }}x_{i}sin\left(\sqrt{\left|x_{i}\right|}\right)$	1000	[−500, 500]	0
F8	RASTRIGIN FUNCTION	$f\left(x\right)=\sum_{i=1}^{n}\left[x_{i}^{2}-10\cos\left(2\pi x_{i}\right)+10\right]$	1000	[−5.12, 5.12]	0
F9	ACKLEY FUNCTION	$f\left(x\right)=-20exp\left(-0.2\sqrt{\frac{1}{n}\overset{n}{\underset{i=1}{\sum }}x_{i}^{2}}\right)-exp\left(\frac{1}{n}\overset{n}{\underset{i=1}{\sum }}\cos\left(2\pi x_{i}\right)\right)+20+e$	1000	[−32, 32]	0
F10	GRIEWANK FUNCTION	$f\left(x\right)=\frac{1}{4000}\overset{n}{\underset{i=1}{\sum }}x_{i}^{2}-\overset{n}{\underset{i=1}{\prod }}\cos\left(\frac{x_{i}}{\sqrt{i}}\right)+1$	1000	[−600, 600]	0
F11	GENERALIZED PENALIZED FUNCTION 1	$\begin{array}{c} f\left(x\right)=\frac{\pi }{n}\left\{10\sin\left(\pi y_{1}\right)+\overset{n-1}{\underset{n=1}{\sum }}{\left(y_{i}-1\right)}^{2}\left[1+10\sin^{2}\left(\pi y_{i+1}\right)\right]+{\left(y_{n}-1\right)}^{2}\right\}\\ +\overset{n}{\underset{i=1}{\sum }}u\left(x_{i},10,100,4\right) \end{array}$ $y_{i}=1+\frac{x_{i}+1}{4}u\left(x_{i},a,k,m\right)=\{\begin{array}{c} k{\left(x_{i}-a\right)}^{m}x_{i}>a\\ 0-a<x_{i}<a\\ k{\left(-x_{i}-a\right)}^{m}x_{i}<-a \end{array}$	1000	[−50, 50]	0
F12	GENERALIZED PENALIZED FUNCTION 2	$\begin{array}{l} f\left(x\right)=0.1\{\sin^{2}\left(3\pi x_{1}\right)+\overset{n}{\underset{i=1}{\sum }}{\left(x_{i}-1\right)}^{2}\left[1+\sin^{2}\left(3\pi x_{i}+1\right)\right]\\ \;+{\left(x_{n}-1\right)}^{2}\left[1+\sin^{2}\left(2\pi x_{n}\right)\right]\}+\overset{n}{\underset{i=1}{\sum }}u\left(x_{i},5,100,4\right) \end{array}$	1000	[−5, 5]	0
F13	LEVY FUNCTION	$\begin{array}{c} f\left(x\right)=\sin^{2}\left(\pi \omega_{1}\right)+\overset{d-1}{\underset{i=1}{\sum }}\left(\omega_{i}+1\right)\left[1+10\;sin^{2}\left(\pi \omega_{i}+1\right)\right]\\ +{\left(\omega_{d}-1\right)}^{2}\left[1+\sin^{2}\left(2\pi \omega_{d}\right)\right] \end{array}$	1000	[−10, 10]	0

**Table 3:** Comparison of the optimization results of five algorithms for six functions (500 dimensions).

		F1	F2	F3	F4	F5	F6
GWO	Obtained best solution	2.68 × 10^−9^	4.42 × 10^4^	7.13 × 10^−4^	1.22 × 10^−6^	1.87 × 10^−7^	3.66 × 10^−8^
	Average	1.33 × 10^−5^	1.02 × 10^5^	6.25 × 10	4.97 × 10^2^	7.82 × 10	1.12 × 10^−2^
	Standard deviation	1.21 × 10^−5^	8.34 × 10^4^	2.54 × 10	9.85 × 10	3.65 × 10	1.01 × 10^−2^
	Success rate	100%	0	68%	52%	76%	96%
BOA	Obtained best solution	4.83 × 10^−15^	8.46 × 10^−17^	1.15 × 10^−18^	6.18 × 10^−6^	2.47 × 10^−11^	1.07 × 10^−12^
	Average	1.28 × 10^−11^	1.27 × 10^−11^	6.26 × 10^−9^	4.98 × 10^2^	1.22 × 10^2^	6.48 × 10^−4^
	Standard deviation	3.04 × 10^−11^	2.33 × 10^−11^	5.32 × 10^−9^	1.88 × 10^2^	7.48 × 10	4.78 × 10^−4^
	Success rate	100%	100%	100%	48%	44%	92%
MPA	Obtained best solution	1.01 × 10^−19^	1.41 × 10^−7^	5.51 × 10^−8^	5.25 × 10^−6^	1.70 × 10^−7^	5.33 × 10^−10^
	Average	5.64 × 10^−16^	2.42 × 10^3^	2.41 × 10^−5^	4.96 × 10^2^	5.91 × 10	1.13 × 10^−3^
	Standard deviation	4.23 × 10^−16^	5.35 × 10^2^	4.29 × 10^−5^	3.12 × 10	8.99	3.89 × 10^−4^
	Success rate	100%	36%	100%	82%	88%	96%
COOT	Obtained best solution	5.82 × 10^−46^	2.10 × 10^−54^	8.47 × 10^−22^	5.97 × 10^−9^	1.86 × 10^−8^	5.94 × 10^−7^
	Average	4.33 × 10^−44^	1.45 × 10^−42^	3.42 × 10^−18^	4.98 × 10^2^	7.42 × 10	1.86 × 10^−3^
	Standard deviation	3.89 × 10^−44^	2.19 × 10^−42^	3.13 × 10^−18^	8.48 × 10	2.67 × 10	6.64 × 10^−3^
	Success rate	100%	100%	100%	88%	92%	96%
DAEMPSO	Obtained best solution	1.11 × 10^−95^	6.67 × 10^−73^	2.00 × 10^−45^	7.73 × 10^−10^	7.90 × 10^−9^	6.66 × 10^−14^
	Average	6.51 × 10^−87^	4.79 × 10^−66^	5.35 × 10^−41^	2.75 × 10^−2^	1.91 × 10^−1^	5.58 × 10^−5^
	Standard deviation	4.45 × 10^−87^	6.33 × 10^−66^	7.33 × 10^−41^	5.34 × 10^−2^	1.56 × 10^−1^	5.33 × 10^−5^
	Success rate	100%	100%	100%	88%	92%	96%

**Table 4:** Comparison of the optimization results of five algorithms for seven test functions (500 dimensions).

		F7	F8	F9	F10	F11	F12	F13
GWO	Obtained best solution	8.38 × 10^4^	1.77 × 10^−8^	6.22 × 10^−9^	9.25 × 10^−12^	2.81 × 10^−10^	9.17 × 10^−7^	8.19 × 10^−8^
	Average	1.41 × 10^5^	3.31 × 10	1.55 × 10^−4^	1.13 × 10^−6^	6.29 × 10^−1^	4.23 × 10	3.79 × 10
	Standard deviation	5.99 × 10^4^	2.59 × 10	3.34 × 10^−4^	5.34 × 10^−7^	2.45 × 10^−1^	9.38	1.42 × 10
	Success rate	0	84%	92%	100%	92%	76%	80%
BOA	Obtained best solution	9.96 × 10^4^	1.82 × 10^−18^	5.12 × 10^−13^	7.02 × 10^−18^	1.16 × 10^−12^	9.89 × 10^−11^	9.12 × 10^−8^
	Average	1.90 × 10^5^	9.09 × 10^−13^	5.47 × 10^−9^	1.46 × 10^−11^	1.14	4.99 × 10	4.58 × 10
	Standard deviation	6.34 × 10^4^	7.16 × 10^−13^	3.67 × 10^−9^	6.55 × 10^−11^	1.06	1.94 × 10	1.05 × 10
	Success rate	0	100%	100%	100%	84%	88%	88%
MPA	Obtained best solution	7.45 × 10^4^	1.82 × 10^−16^	2.53 × 10^−15^	7.34 × 10^−20^	8.48 × 10^−9^	9.68 × 10^−12^	7.63 × 10^−12^
	Average	1.17 × 10^5^	9.09 × 10^−13^	1.53 × 10^−9^	1.11 × 10^−16^	2.18 × 10^−1^	4.56 × 10	3.20 × 10
	Standard deviation	6.44 × 10^4^	3.48 × 10^−13^	1.70 × 10^−9^	7.09 × 10^−16^	3.85 × 10^−1^	1.76 × 10	9.16
	Success rate	0	100%	100%	100%	92%	88%	92%
COOT	Obtained best solution	1.15 × 10^5^	3.82 × 10^−14^	9.32 × 10^−19^	1.69 × 10^−14^	3.54 × 10^−10^	1.19 × 10^−12^	8.78 × 10^−11^
	Average	1.35 × 10^5^	1.45 × 10^−11^	8.88 × 10^−16^	7.21 × 10^−11^	2.17 × 10^−1^	5.50 × 10	3.96 × 10
	Standard deviation	4.93 × 10^4^	2.76 × 10^−11^	4.27 × 10^−16^	2.71 × 10^−11^	9.80 × 10^−2^	3.24 × 10	3.32 × 10
	Success rate	0	100%	100%	100%	96%	88%	92%
DAEMPSO	Obtained best solution	6.69 × 10^−13^	1.82 × 10^−17^	4.85 × 10^−23^	2.62 × 10^−19^	5.86 × 10^−14^	4.31 × 10^−16^	5.12 × 10^−7^
	Average	2.31 × 10	9.09 × 10^−13^	8.88 × 10^−16^	7.77 × 10^−16^	1.75 × 10^−4^	6.21 × 10^−7^	3.84 × 10^−4^
	Standard deviation	1.03 × 10	1.85 × 10^−13^	6.58 × 10^−16^	4.72 × 10^−16^	5.70 × 10^−4^	1.38 × 10^−7^	7.81 × 10^−4^
	Success rate	36%	100%	100%	100%	96%	100%	96%

**Table 5:** Comparison of the optimization results of five algorithms for six test functions (800 dimensions).

		F1	F2	F3	F4	F5	F6
GWO	Obtained best solution	6.18 × 10^−9^	1.02 × 10^5^	7.13 × 10^−7^	5.11 × 10^−12^	7.25 × 10^−8^	8.20 × 10^−8^
	Average	8.36 × 10^−5^	2.70 × 10^5^	6.70 × 10	7.97 × 10^2^	1.44 × 10^2^	2.49 × 10^−2^
	Standard deviation	1.36 × 10^−5^	3.59 × 10^3^	8.24	1.35 × 10	2.97 × 10	1.12 × 10^−2^
	Success rate	100%	0	60%	52%	64%	92%
BOA	Obtained best solution	3.90 × 10^−15^	6.21 × 10^−17^	5.06 × 10^−15^	1.68 × 10^−12^	4.27 × 10^−11^	7.81 × 10^−9^
	Average	1.28 × 10^−11^	1.28 × 10^−11^	5.68 × 10^−9^	7.98 × 10^2^	1.97 × 10^2^	6.88 × 10^−4^
	Standard deviation	1.21 × 10^−11^	6.02 × 10^−11^	5.35 × 10^−9^	1.62 × 10^2^	8.89 × 10	8.23 × 10^−4^
	Success rate	100%	100%	100%	68%	80%	96%
MPA	Obtained best solution	6.04 × 10^−18^	4.11 × 10^−6^	1.55 × 10^−14^	5.99 × 10^−12^	2.10 × 10^−11^	1.52 × 10^−8^
	Average	5.01 × 10^−15^	5.08 × 10^3^	4.02 × 10^−5^	7.95 × 10^2^	1.24 × 10^2^	1.40 × 10^−3^
	Standard deviation	9.73 × 10^−15^	4.62 × 10^3^	6.76 × 10^−5^	5.73 × 10^2^	9.73 × 10	9.79 × 10^−3^
	Success rate	100%	60%	88%	64%	56%	92%
COOT	Obtained best solution	2.58 × 10^−56^	2.10 × 10^−64^	4.78 × 10^−22^	7.59 × 10^−12^	6.81 × 10^−8^	4.59 × 10^−14^
	Average	5.92 × 10^−51^	1.27 × 10^−53^	1.85 × 10^−17^	6.36 × 10^3^	1.46 × 10^2^	2.91 × 10^−3^
	Standard deviation	4.40 × 10^−50^	6.48 × 10^−51^	9.56 × 10^−17^	2.74 × 10^3^	1.07 × 10^2^	6.30 × 10^−3^
	Success rate	100%	100%	100%	48%	68%	96%
DAEMPSO	Obtained best solution	1.88 × 10^−97^	3.55 × 10^−67^	2.63 × 10^−45^	5.13 × 10^−11^	9.70 × 10^−13^	3.66 × 10^−9^
	Average	4.37 × 10^−90^	2.41 × 10^−61^	1.44 × 10^−36^	1.05 × 10^−1^	1.11 × 10^−4^	4.11 × 10^−5^
	Standard deviation	8.94 × 10^−88^	8.15 × 10^−60^	3.90 × 10^−36^	9.52 × 10^−2^	2.63 × 10^−5^	2.23 × 10^−5^
	Success rate	100%	100%	100%	72%	96%	96%

**Table 6:** Comparison of the optimization results of five algorithms for seven test functions (800 dimensions).

		F7	F8	F9	F10	F11	F12	F13
GWO	Obtained best solution	1.92 × 10^5^	1.13 × 10^−8^	8.81 × 10^−9^	2.95 × 10^−12^	6.81 × 10^−8^	1.79 × 10^−7^	9.18 × 10^−8^
	Average	2.45 × 10^5^	7.15 × 10	1.30 × 10^−3^	4.23 × 10^−2^	7.02 × 10^−1^	7.17 × 10	6.41 × 10
	Standard deviation	7.09 × 10^4^	4.58 × 10	6.19 × 10^−4^	9.58 × 10^−3^	5.19 × 10^−1^	3.55 × 10	2.99 × 10
	Success rate	0	80%	96%	96%	92%	88%	84%
BOA	Obtained best solution	2.06 × 10^5^	2.18 × 10^−16^	2.15 × 10^−19^	7.52 × 10^−16^	6.11 × 10^−8^	4.55 × 10^−11^	9.55 × 10^−11^
	Average	3.15 × 10^5^	9.09 × 10^−13^	2.22 × 10^−14^	1.47 × 10^−11^	1.14	7.99 × 10	7.31 × 10
	Standard deviation	2.01 × 10^5^	4.48 × 10^−13^	5.20 × 10^−14^	3.83 × 10^−11^	8.05 × 10^−1^	5.62 × 10	6.74 × 10
	Success rate	0	100%	100%	100%	92%	76%	84%
MPA	Obtained best solution	1.65 × 10^5^	3.52 × 10^−15^	1.50 × 10^−15^	4.69 × 10^−22^	2.48 × 10^−9^	6.89 × 10^−9^	8.18 × 10^−8^
	Average	2.09 × 10^5^	1.82 × 10^−12^	1.81 × 10^−9^	1.12 × 10^−16^	3.60 × 10^−1^	7.68 × 10	6.01 × 10
	Standard deviation	1.95 × 10^5^	6.33 × 10^−13^	5.05 × 10^−9^	9.27 × 10^−17^	2.23 × 10^−1^	3.78 × 10	4.13 × 10
	Success rate	0	100%	100%	100%	92%	80%	84%
COOT	Obtained best solution	1.98 × 10^5^	3.51 × 10^−17^	5.48 × 10^−18^	4.12 × 10^−19^	8.15 × 10^−9^	2.26 × 10^−12^	8.25 × 10^−11^
	Average	2.54 × 10^5^	9.09 × 10^−13^	2.22 × 10^−14^	5.66 × 10^−15^	5.66 × 10^−1^	7.98 × 10	6.74 × 10
	Standard deviation	9.25 × 10^4^	7.88 × 10^−13^	5.91 × 10^−14^	3.75 × 10^−15^	4.89 × 10^−1^	5.09 × 10	4.90 × 10
	Success rate	0	100%	100%	100%	92%	84%	88%
DAEMPSO	Obtained best solution	6.94 × 10^−8^	7.50 × 10^−17^	5.51 × 10^−21^	7.25 × 10^−18^	3.65 × 10^−13^	2.35 × 10^−12^	3.28 × 10^−9^
	Average	3.48	9.09 × 10^−13^	8.88 × 10^−16^	3.33 × 10^−16^	2.99 × 10^−7^	6.42 × 10^−6^	4.50 × 10^−2^
	Standard deviation	2.84	9.24 × 10^−13^	7.41 × 10^−16^	9.13 × 10^−16^	1.35 × 10^−7^	4.36 × 10^−6^	2.03 × 10^−2^
	Success rate	44%	100%	100%	92%	100%	100%	96%

**Table 7:** Comparison of the optimization results of five algorithms for six test functions (1000 dimensions).

		F1	F2	F3	F4	F5	F6
GWO	Obtained best solution	2.75 × 10^−6^	5.93 × 10^4^	3.55 × 10^−2^	2.61 × 10^−2^	3.43 × 10^−3^	8.25 × 10^−6^
	Average	6.68 × 10^−4^	1.02 × 10^5^	7.13	5.97 × 10^2^	1.87 × 10^2^	3.03 × 10^−2^
	Standard deviation	5.39 × 10^−4^	9.48 × 10^4^	5.87	2.54 × 10^2^	1.15 × 10^2^	5.02 × 10^−2^
	Success rate	100%	0%	88%	36%	44%	88%
BOA	Obtained best solution	7.15 × 10^−13^	4.45 × 10^−12^	8.12 × 10^−11^	5.51 × 10^−5^	1.15 × 10^−4^	4.65 × 10^−6^
	Average	1.29 × 10^−11^	1.26 × 10^−11^	6.05 × 10^−9^	6.18 × 10^2^	2.47 × 10^2^	1.83 × 10^−1^
	Standard deviation	8.01 × 10^−12^	1.30 × 10-^−12^	5.83 × 10^−9^	3.90 × 10^2^	8.94 × 10	1.10 × 10^−1^
	Success rate	100%	100%	100%	32%	44%	84%
MPA	Obtained best solution	6.15 × 10^−18^	7.64 × 10^−8^	9.76 × 10^−7^	3.65 × 10^−6^	4.38 × 10^−4^	2.19 × 10^−8^
	Average	1.01 × 10^−14^	1.41 × 10^−4^	5.51 × 10^−4^	5.95 × 10^2^	1.70 × 10^2^	1.36 × 10^−1^
	Standard deviation	5.71 × 10^−14^	7.95 × 10^−5^	1.46 × 10^−4^	2.41 × 10^2^	1.25 × 10^2^	5.29 × 10^−1^
	Success rate	100%	100%	100%	44%	48%	92%
COOT	Obtained best solution	9.69 × 10^−36^	7.52 × 10^−39^	2.66 × 10^−29^	2.41 × 10^−7^	7.27 × 10^−9^	3.65 × 10^−10^
	Average	5.82 × 10^−26^	2.10 × 10^−34^	8.47 × 10^−22^	5.97 × 10^2^	1.86 × 10^2^	5.94 × 10^−4^
	Standard deviation	5.59 × 10^−26^	6.84 × 10^−34^	3.36 × 10^−22^	2.86 × 10^2^	1.37 × 10^2^	1.44 × 10^−4^
	Success rate	100%	100%	100%	48%	48%	96%
DAEMPSO	Obtained best solution	1.26 × 10^−82^	3.92 × 10^−62^	1.34 × 10^−48^	6.16 × 10^−9^	9.38 × 10^−10^	7.46 × 10^−6^
	Average	1.11 × 10^−77^	6.67 × 10^−57^	2.00 × 10^−35^	3.15 × 10^−1^	7.90 × 10^−1^	6.66 × 10^−2^
	Standard deviation	7.46 × 10^−76^	6.10 × 10^−57^	2.76 × 10^−35^	1.43 × 10^−1^	4.95 × 10^−1^	3.11 × 10^−2^
	Success rate	100%	100%	100%	84%	88%	92%

**Table 8:** Comparison of the optimization results of five algorithms for seven test functions (1000 dimensions).

		F7	F8	F9	F10	F11	F12	F13
GWO	Obtained best solution	2.26 × 10^5^	3.46 × 10^−6^	2.72 × 10^−9^	9.47 × 10^−7^	4.71 × 10^−8^	4.79 × 10^−8^	4.68 × 10^−11^
	Average	3.13 × 10^5^	1.33 × 10^2^	3.13 × 10^−3^	9.25 × 10^−2^	8.13 × 10^−1^	9.17 × 10	8.19 × 10
	Standard deviation	1.96 × 10^5^	1.12 × 10^2^	2.23 × 10^−3^	5.63 × 10^−2^	5.05 × 10^−1^	7.15 × 10	5.36 × 10
	Success rate	0	32%	88%	92%	96%	76%	80%
BOA	Obtained best solution	1.37 × 10^4^	7.16 × 10^−15^	9.49 × 10^−19^	5.68 × 10^−14^	5.19 × 10^−5^	2.29 × 10^−6^	8.83 × 10^−8^
	Average	3.97 × 10^5^	1.82 × 10^−12^	5.12 × 10^−9^	1.41 × 10^−11^	1.16	9.89 × 10	9.12 × 10
	Standard deviation	1.84 × 10^5^	4.45 × 10^−12^	3.10 × 10^−9^	1.65 × 10^−11^	9.49 × 10^−1^	4.12 × 10	2.34 × 10
	Success rate	0	100%	100%	100%	96%	88%	88%
MPA	Obtained best solution	1.01 × 10^5^	5.99 × 10^−19^	8.11 × 10^−15^	7.00 × 10^−18^	3.31 × 10^−10^	6.39 × 10^−8^	9.77 × 10^−5^
	Average	2.86 × 10^5^	1.32 × 10^−13^	2.53 × 10^−9^	1.11 × 10^−16^	4.48 × 10^−1^	9.68 × 10	7.63 × 10
	Standard deviation	1.34 × 10^5^	6.73 × 10^−13^	1.06 × 10^−9^	4.95 × 10^−16^	3.34 × 10^−1^	6.38 × 10	6.06 × 10
	Success rate	0	100%	100%	100%	84%	88%	88%
COOT	Obtained best solution	2.88 × 10^5^	8.50 × 10^−18^	4.92 × 10^−18^	6.50 × 10^−15^	6.68 × 10^−5^	6.72 × 10^−6^	1.82 × 10^−11^
	Average	3.40 × 10^5^	3.82 × 10^−11^	9.32 × 10^−14^	1.69 × 10^−14^	5.54 × 10^−1^	1.19 × 10^2^	8.78 × 10
	Standard deviation							
	Success rate	0	100%	100%	100%	88%	72%	84%
DAEMPSO	Obtained best solution	8.85 × 10^−7^	4.90 × 10^−15^	1.99 × 10^−25^	8.34 × 10^−22^	5.54 × 10^−10^	1.29 × 10^−9^	7.65 × 10^−4^
	Average	1.14 × 10	1.82 × 10^−12^	8.88 × 10^−16^	2.22 × 10^−2^	5.86 × 10^−7^	4.31 × 10^−2^	5.12 × 10^−3^
	Standard deviation	1.04 × 10	7.96 × 10^−13^	6.04 × 10^−16^	1.92 × 10^−2^	4.99 × 10^−7^	3.67 × 10^−2^	4.98 × 10^−3^
	Success rate	32%	100%	100%	92%	100%	92%	96%

## Data Availability

Test function source: http://www.sfu.ca/~ssurjano/index.html (accessed on 4 May 2021).
